# Trust, entrustment decisions and a few things we shouldn’t forget

**DOI:** 10.1007/s40037-017-0337-1

**Published:** 2017-03-10

**Authors:** Marjan J. B. Govaerts

**Affiliations:** Department of Educational Development and Research, FHML, Maastricht University, Maastricht, The Netherlands

For many decades, medical educators have been struggling with assessment, as illustrated by the predominance of assessment in medical education research as well as the ongoing profound and sometimes heated discussions about how to best assess trainees’ competence and development [1–3]. In any event, efforts to change assessment systems are oriented towards the goal of improving health care quality by improving medical education, assessment and trainee learning.

The rise of outcome-based models of education and increasing pressures for educational and professional accountability resulted in a growing emphasis on assessments that provide more direct evidence of the ultimate proficiencies of interest, i. e. performance in practice. As a consequence, medical education has witnessed radical changes in assessment approaches, including development and implementation of competency frameworks and, more recently, the concept of Entrustable Professional Activities (EPAs). In workaday reality of medical training EPAs, trust and entrustment seem to emerge as concepts that are more intuitive and meaningful than competencies. They are readily embraced by clinicians and medical educators as well, suggesting we may finally have found the Holy Grail in assessment [4, 5]. Part of the appeal of the entrustment concept appears to lie in the fact that entrustment decisions and the inherent ‘willingness to take risk’ align with the reality of medical practice: this is what clinicians do, day after day [4]. However, as persuasively argued by Holmboe and Batalden, achieving the desired transformation in medical education and health care may require disruptive change and moving forward to training models that are definitely less aligned to current reality and thus difficult to implement [6]. The pivotal question, then, seems to be when and how the adoption of trust and entrustment as core values in supervision and assessment supports achievement of desired transformations in health care education and practice. What are the things we should not forget?

In this issue of Perspectives on Medical Education, the paper by Holzhausen, Maaz, Cianciolo, Ten Cate and Peters presents a conceptual framework of entrustment decisions, drawing from work in domains of organizational psychology and military psychology [7]. It aims to advance our understanding of how (ad-hoc) entrustment decisions are made in day-to-day clinical practice. The proposed conceptual framework clearly illustrates the complexity of the entrustment decision-making process in health care settings: entrustment is time, task and context dependent and influenced by factors related to the trustee and trustor and their working relationship. Although quite implicitly, the conceptual model also shows that entrustment decisions need to be grounded in high-quality assessments of trainee competence. It goes without any doubt that quality of entrustment decisions is inextricably linked to a supervisor’s competence in assessment. Beliefs about trust and entrustment being intuitive and aligned to the reality of day-to-day medical practice may imply that clinical experience and/or experience in supervision of trainees ensures entrustment decisions to be trustworthy. Although experience, without any doubt, is crucial in competence development, ‘time-on-task’ in itself does not produce expertise, neither in patient care [8] nor in performance assessment [9]. If we want high-quality entrustment decisions for high-quality care, we need to support our supervisors in developing the necessary professional expert judgement. Obviously, development of professional judgement requires education (e. g. training, workshops), but it first and foremost requires deliberation and deliberate practice. Brief, one-off training sessions will no longer do; as in any other domain, development of professional judgement in assessment requires long-term support, coaching and feedback as well as critical reconsideration and reconstruction of (one’s own) professional practice in supervision and competence assessment [10, 11]. This may require fundamentally different and less familiar approaches to faculty development.

In line with findings from research on performance assessments in medicine as well as other domains such as organizational psychology, the conceptual model furthermore implies that entrustment decisions are influenced by political, financial and cultural (norms and values) factors in the organizational/educational context. This implies that we have to look beyond entrustment in the dyadic trainee-supervisor relationship. There is a wealth of research findings showing that exactly the culture (i. e. ‘the way we do things around here’) and contextual factors as described above may interfere with assessment quality. In medicine, for example, research findings clearly show that cultural values of autonomy in learning and practice, as well as aims to maximize efficiency in delivery of health care services, conflict with a culture that values direct observation or documentation of meaningful performance data [6, 12, 13]. In fact, cultural context may be the key factor determining trustworthiness of entrustment decisions. Building our assessment systems on concepts of trust and entrustment may thus require a shift in educational culture and perspectives leading to fundamental changes in the way we organize medical training and supervision. A critical first step might be to ensure that trainees and supervisors are able to engage in extended and trusting working relationships that not only facilitate direct observation and assessment, but also provide opportunities as well as challenge trainees and supervisors to co-configure the learning and assessment processes embedded in their work, in order to maximally tailor trust and supervision to trainee needs as well as patient safety.

Actually, trust research in organizational psychology typically envisions trust as a substitute for control, reducing the need to monitor behaviours and emphasizing efficiency in organizational performance. From this perspective, our focus on trust and entrustment decisions seems to meet with current demands in health care to increase efficiency in attempts to improve quality and safety while reducing cost. Trust, in other words, fits approaches to health care delivery that focus on ‘execution-as-efficiency’ [14]. The essence of commitment to improvement and excellence, however, is commitment to *learning *[15]. Transformation of medical training to meet challenges of rapidly and substantially changing health care systems may thus require a shift in organizational mindset from ‘execution as efficiency’ towards ‘execution-as-learning’ [14]. This implies paying attention to a concept which is closely related, but conceptually different from trust, i. e. psychological safety. Whereas trust focuses on judgements about trustworthiness of *others*, psychological safety is about acknowledging that it takes time to learn, about seeing tasks as opportunities for learning, as well as about willingness to take the risk of speaking up and raising questions and concerns. Psychological safety implies a culture that values deliberation and collaboration – rather than decisiveness and efficiency [14]. Research findings in organizational psychology show that psychologically safe work and learning environments enable help and feedback seeking as well as speaking up about errors and concerns. Psychological safety equally facilitates provision of honest – and sometimes tough – feedback [14]. Psychological safety may thus be a necessary condition to foster a culture in which high-quality entrustment decisions support high-quality patient care as well as learning and continuous performance improvement.

Transforming our assessment systems to foster excellence in patient care thus requires efforts that should not be underestimated, even – or maybe exactly – when building our assessments on seemingly intuitive concepts such as trust.
